# Gold Nanorods as a Contrast Agent for Doppler Optical Coherence Tomography

**DOI:** 10.1371/journal.pone.0090690

**Published:** 2014-03-03

**Authors:** Bo Wang, Larry Kagemann, Joel S. Schuman, Hiroshi Ishikawa, Richard A. Bilonick, Yun Ling, Ian A. Sigal, Zach Nadler, Andrew Francis, Michelle G. Sandrian, Gadi Wollstein

**Affiliations:** 1 UPMC Eye Center, Eye and Ear Institute, Ophthalmology and Visual Science Research Center, Department of Ophthalmology, University of Pittsburgh School of Medicine, Pittsburgh, Pennsylvania, United States of America; 2 Department of Bioengineering, Swanson School of Engineering, University of Pittsburgh, Pittsburgh, Pennsylvania, United States of America; 3 Department of Biostatistics, Graduate School of Public Health, University of Pittsburgh, Pittsburgh, Pennsylvania, United States of America; 4 Medical Scientist Training Program, Carnegie Mellon University and University of Pittsburgh, Pittsburgh, Pennsylvania, United States of America; Bascom Palmer Eye Institute, University of Miami School of Medicine, United States of America

## Abstract

**Purpose:**

To investigate gold nanorods (GNRs) as a contrast agent to enhance Doppler optical coherence tomography (OCT) imaging of the intrascleral aqueous humor outflow.

**Methods:**

A serial dilution of GNRs was scanned with a spectral-domain OCT device (Bioptigen, Durham, NC) to visualize Doppler signal. Doppler measurements using GNRs were validated using a controlled flow system. To demonstrate an application of GNR enhanced Doppler, porcine eyes were perfused at constant pressure with mock aqueous alone or 1.0×10^12^ GNR/mL mixed with mock aqueous. Twelve Doppler and volumetric SD-OCT scans were obtained from the limbus in a radial fashion incremented by 30°, forming a circular scan pattern. Volumetric flow was computed by integrating flow inside non-connected vessels throughout all 12 scans around the limbus.

**Results:**

At the GNR concentration of 0.7×10^12^ GNRs/mL, Doppler signal was present through the entire depth of the testing tube without substantial attenuation. A well-defined laminar flow profile was observed for Doppler images of GNRs flowing through the glass capillary tube. The Doppler OCT measured flow profile was not statistically different from the expected flow profile based upon an autoregressive moving average model, with an error of −0.025 to 0.037 mm/s (p = 0.6435). Cross-sectional slices demonstrated the ability to view anterior chamber outflow ex-vivo using GNR-enhanced Doppler OCT. Doppler volumetric flow measurements were comparable to flow recorded by the perfusion system.

**Conclusions:**

GNRs created a measureable Doppler signal within otherwise silent flow fields in OCT Doppler scans. Practical application of this technique was confirmed in a constant pressure ex-vivo aqueous humor outflow model in porcine eyes.

## Introduction

The ability to image flow, such as using Doppler ultrasound and laser flowtometry, has impacted our understanding of physiology [1] and medical management [2], [3]. However, the ability to image low volume flow fields with existing technology is still limited. It would be advantageous if these flow fields could be made visible, facilitating measurement, without perturbation. Doppler ultrasound is limited by its resolution, which dependent on the frequency of the ultrasound pulse. Even high frequency ultrasound can only achieve lateral and axial resolution of ∼50 µm [4]. Meanwhile, laser flowtometry lacks the ability to measure depth information regarding the outflow vessels of interest [5].

Optical coherence tomography (OCT) is an imaging technique that creates high-resolution structural scans of optically reflecting tissues using low-coherence interferometry [6]. Advances in acquisition time makes OCT a practical device for detecting flows [7]. OCT's ability to detect flow with high spatial resolution had been demonstrated in a variety of tissues, including the human retina [8], skin [9], and the heart [10].

As with other Doppler imaging technologies, only fluids containing scattering media at the wavelength of the OCT light source can exhibit Doppler shifts. In some instances physiologically relevant flows, such as that of the aqueous humor, do not show a distinct Doppler signal. Aqueous humor has particular relevance to glaucoma because the balance between its production and outflow determined intraocular pressure (IOP). Elevated IOP had been demonstrated to be the main risk factor for the development of glaucoma, an optic neuropathy that is a leading cause of irreversible blindness worldwide [11]. While not useful for assessing IOP, the addition of GNRs to the outflow pathway may allow visualization of Doppler signal within the normally silent aqueous outflow pathways. The ability to image aqueous outflow could improve our understanding of outflow patterns and glaucoma pathophysiology.

Nanoparticles have been increasingly used as a structural [12] and functional [13], [14] contrast agent in branches of medicine. We have previously demonstrated the ability to enhance structural OCT signals using intravitreal GNR injections [15]. However, despite the use of GNRs [12], [15], [16] and other contrast agents [17]–[19] to enhance structural OCT imaging, little work had been done to explore their ability to enhance functional measurements such as Doppler. This manuscript describes the use of GNRs as a contrast agent for Doppler OCT that allows ex-vivo imaging of the outflow pathway of porcine eyes.

## Methods

### Titration

A concentrated polyelectrolyte sodium salt-coated plasmon resonant GNR with a localized surface plasmon resonance of 850 nm and aspect ratio of 4 (∼40 nm×10 nm) were created using a seed-mediated, surfactant directed wet chemical synthesis (Nanorods, Germantown, MD). The polystyrene sulfonate (PSS) coating was used to neutralize the positively charged surfactants used in GNR synthesis. [15] The GNRs were titrated with deionized water to 5 different concentrations: 0.3, 0.4, 0.5, 0.7 and 1.0×10^12^ GNRs/mL. The addition of water or mock aqueous does not alter the spectrophotometry profile of the GNRs (Figure S1). Doppler signal was subjectively assessed in a glass capillary tube of radius 0.32 mm using the different dilutions. The tube size was chosen to maximize our ability to see Doppler flow throughout a region that would be larger than any aqueous channel.

### OCT scanning

Doppler scans (2×3×2 mm, 700×20×1024 pixels) were acquired using spectral-domain (SD-) OCT (Bioptigen, Durham, NC) while the fluid was stationary within the capillary tube. The OCT had a 100 nm bandwidth light source centered on 870 nm, yielding a theoretical axial resolution of 3.5 µm. 12 Doppler frames were acquired with an acquisition rate of 28 kHz, resulting in a Nyquist limit of 4.62 mm/s normal to the scanning beam. The conversion of OCT Doppler signal to angle-corrected velocity is specified in the supporting materials.

### Validation

A GNR solution mixed with deionized water was scanned as it flowed through the capillary tube. The glass tube was suspended 45 degrees to the direction of the OCT scanning beam. The GNR solution was pumped at a volumetric rate of 0.56 µL/s with a constant pressure perfusion system and was given 10 minutes to reach a steady state. The rate of perfusion was recorded using a DATAQ acquisition unit (DATAQ Instruments, Akron, OH) and Doppler scans (2×0.4×2 mm, 700×20×1024 pixels) were acquired using SD-OCT. The flow profile recorded by Doppler OCT was compared to a theoretical flow profile assuming laminar flow given a volumetric flow rate of 0.56 µL/s. The two measurements were compared using an autoregressive-moving-average (ARMA) model. A negative log difference was used to reduce the expected autocorrelation between flow values in adjacent pixels.

### Ex-vivo porcine model

We used ethically sourced ex-vivo porcine eyes from the local abattoir (Thoma's Meat Market, 748 Dinnerbell Road, Saxonburg, PA 16056). Permission was obtained from the abattoir to use the animal parts for research. Therefore, no IACUC approval was required for these experiments. Two cohorts of ex-vivo porcine eyes were warmed to 40°C and perfused in the anterior chamber with (1) Barany's mock aqueous [20] alone (control; n = 6) or (2) 1×10^12^ GNRs/mL solution with mock aqueous (n = 2). The eyes were scanned within 16 hours of death. A mix of GNR and mock aqueous was tested using spectrophotometry to insure that the functional properties of the nanorods were not altered.

**Figure 1:**
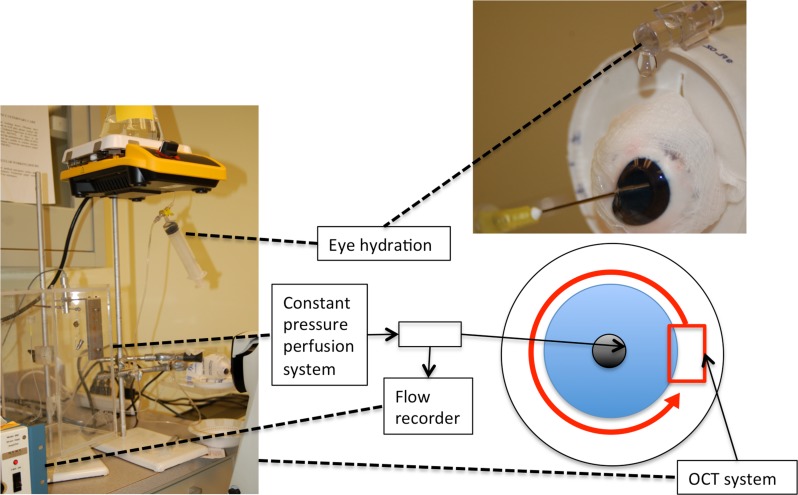
Experimental setting for ex-vivo imaging of anterior chamber outflow in porcine eyes. A needle attached to a constant pressure perfusion system, whose flow is measured by the flow recorder, is inserted between the lens and iris in the posterior chamber. 12 OCT scans (red box) were taken along the limbus of the eye(red arrow). Dashed lines shows the equipment indicated by the boxes.

A 27-gauge perfusion needle was inserted through the cornea, between the lens and the iris into the posterior chamber (Figure 1). The site of insertion of the needle was to the nasal side for all eyes. A trial eye demonstrated that injecting GNR solution alone was not sufficient to image the outflow system because the mixture of the injected GNR with aqueous humor decreased GNR concentration. Therefore, a 30-gauge needle was inserted to the temporal side to withdraw aqueous humor during initial transfer of GNRs solution. GNR solutions flowed under constant pressure for 10 minutes to allow outflow to stabilize to a constant (Figure S2). Doppler scans were taken over a 4×4×2 mm area (700×20×1024 pixels) around the anterior chamber of the eye. Twelve Doppler (700×20×1024 pixels) and volumetric scans (one per clock hour) were taken around the limbus with a scan size of 4×4×2 mm. Scans were oriented in a radial fashion incremented by 30° from scan to scan.

Volumetric flow rates were calculated by integrating all flow in a circular pattern around the limbus. Individual non-connected vessels were identified on 3D structural scans. The angle between the scanning beam and vessel was computed from a vector formed by vessel centroids as seen on structural images. The angle was used to calculate the angle-corrected velocity. The volumetric flow through the vessel was determined by integrating the angle-corrected velocity and over the region of the vessel. Total volumetric flow was calculated as the sum of observed flow around the limbus by a masked observer.

## Results

### Titration

**Figure 2:**
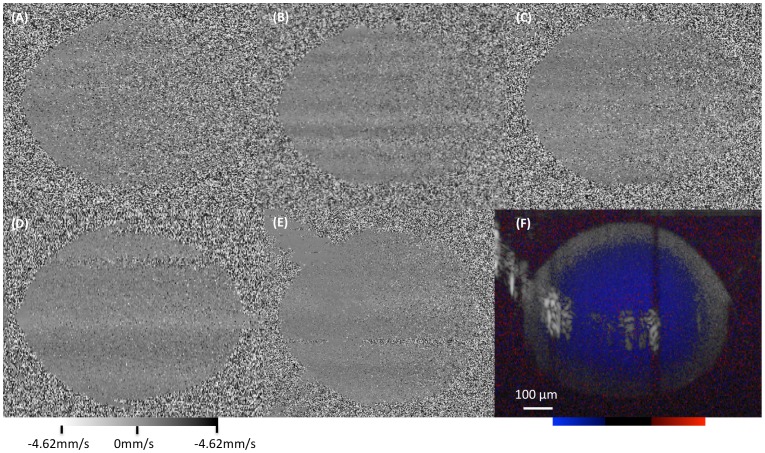
Determining optimal GNR concentration. (A–E) Doppler OCT cross-sectional images of non-flowing gold nanorods (GNR) at (A) 0.3, (B) 0.4, (C) 0.5, (D) 0.7 and (E) 1.0×10^12^ GNRs/mL. The grayscale mapping represents the detected flow towards or away from the OCT scanning beam, centered on 0 mm/s (gray). (F) GNR enhanced saline flowing in a glass tube with a concentration of 1.0×10^12^ GNRs/mL. The color map represents flow towards (red), away (blue) and stationary (black) relative to the OCT scanning beam. (D–F) shows the ability to get Doppler signal with a tube radius of up to 0.32 mm. The red circle corresponds to the outline of the inner surface of the tube. Blue and red corresponded to flow away and towards the probe, respectively. The area of hyper reflective convex arch within F is an imaging artifact.

Doppler signal was observed in the testing tube across all GNRs dilutions (Figure 2). A concentration of 1.0×10^12^ GNRs/mL yielded an optimal Doppler signal with only a moderate increase in Doppler noise as the A-scan penetrates further in the solution, and accordingly this concentration was chosen for all further testing. All stationary Doppler scans showed a systematic alternating pattern in the Doppler signal that was attributed to room or galvonometer vibrations during the scanning procedure.

### Validation

**Figure 3:**
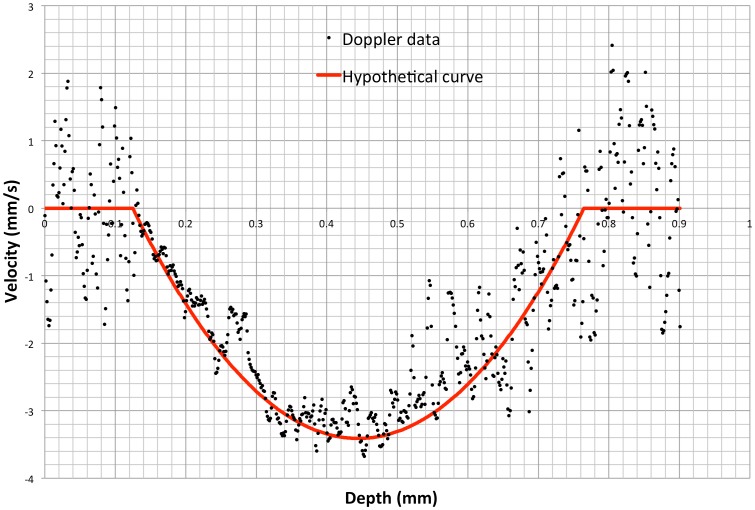
Assessing Doppler velocity measurement. Angle corrected velocity profile acquired within a glass capillary tube containing 1.0×10^12^ gold nanorods/mL with the computed velocity profile overlain in red.

OCT Doppler cross-sectional scans of testing tube with constant flow of 0.56 µL/s with 1.0×10^12^ GNRs/mL solution showed laminar flow (Figure 3A). A theoretical velocity profile within the tube was calculated based upon the flow measured by the perfusion system. The difference between velocity profile assessed by Doppler OCT and the theoretical flow profile given a constant flow of 0.56 µL/s showed no statistically significant difference. The difference was centered at 0.192±0.498 mm/s with an ARMA intercept ranging between −0.025 to 0.037 mm/s (p = 0.6435). A difference between the theoretical and recorded of flow rate whose confidence interval contains 0 indicates that OCT was able to properly measure GNR-enhanced Doppler.

### Ex-vivo porcine model

**Figure 4:**
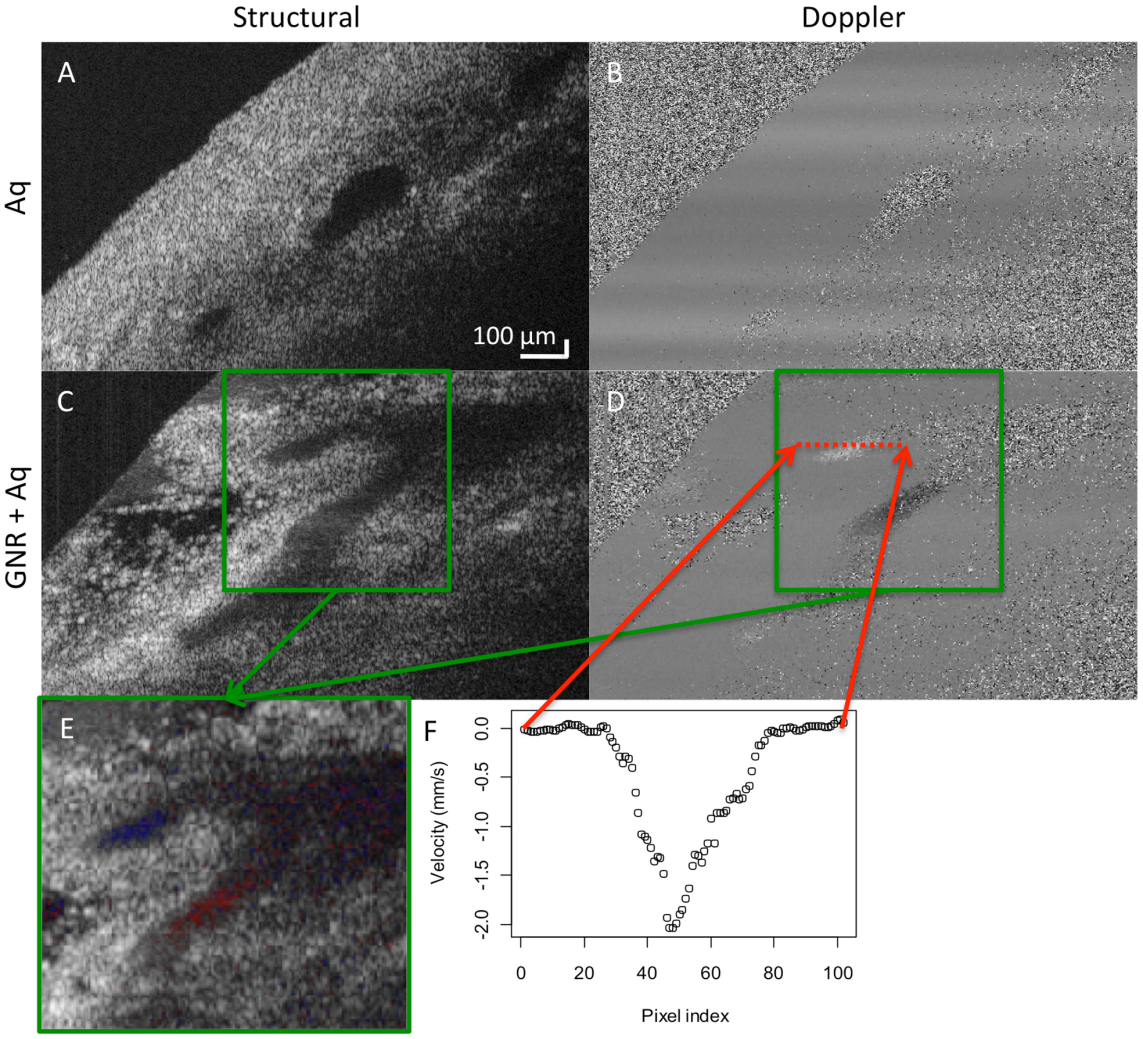
Ex-vivo Doppler imaging. Structural and Doppler OCT scans of the outflow pathway in porcine eyes perfused with (A–B) mock aqueous (control, Aq) and (C–D) 1×10^12^ gold nanorods/mL with mock aqueous (GNR+Aq). (E) Merged structural and Doppler scan corresponding to the location in the green box. Red and blue corresponds to flow away and towards the probe, respectively. (F) Velocity profile of the Doppler scans in the location corresponding to the red dotted lines.

Spectrophotometry reading of GNRs mixed with mock aqueous showed characteristic peaks at 850 nm and 500 nm, similar to the profile obtained from the innate GNR. Using mock aqueous alone, the Doppler signal in the pig anterior chamber was not measurable (Figure 4A). All vessels perfused with mock aqueous appeared dark on the structural scan, showing little reflectivity. The addition of 1.0×10^12^ GNRs/mL solution increased the contrast within the aqueous outflow vessels and showed clear Doppler signals (Figure 4E). Velocity profile of the Doppler scan showed laminar flow within the vessels (Figure 4F). The volumetric flow rate measured by the reservoir system was 3.42 and 7.02 µl/min in each eye. The rate computed by SD-OCT Doppler for the two eyes was 4.41 and 7.31 µl/min, respectively.

## Discussion

This study demonstrated the first use of GNRs for enhancing *functional* ophthalmic imaging using OCT. An optimal concentration of GNRs was identified to maximize penetration without losing signal in a system with fixed flow. The validity of the flow measurement using GNR enhanced Doppler OCT was confirmed in an experiment with known flow parameters. Finally, the use of GNRs and Doppler OCT was demonstrated *ex-vivo* in porcine eyes, providing the first Doppler OCT scans of intrascleral aqueous outflow.

As OCT scanning beam traverse through a medium, scattering and absorption reduced the signal-to-noise ratio of the system. The tradeoff between signal strength and depth of penetration played an important factor in the optimal GNR concentration. Low concentrations of GNR resulted in poor Doppler imaging because it did not induce enough Doppler signal after scattering loss, creating the inhomogeneous Doppler signal (Figure 2A–C). Increasing GNR concentration improved the quality of Doppler OCT. However, structural scanning experienced lower depth of penetration due to increased scattering from the solution. The optimal concentration of GNRs for enhanced Doppler signal without the creation of obstructive shadows was between 0.7×10^12^–1.0×10^12^ GNRs/ml (Figure 2).

We used a controlled flow model to validate the GNR enhanced Doppler signals measurements. Comparison between the computed theoretical velocity profile and Doppler OCT velocity profile showed a normal distribution difference centered around zero, confirming the accuracy of Doppler flow measurements.

We demonstrated GNRs' potential to enhance Doppler OCT signals by using the ex-vivo porcine model (Figure 4). Due to the small size of vessels present in the anterior chamber outflow pathway, we used a 1.0×10^12^ GNRs/mL solution. We expect the solution to mix with the remaining aqueous humor remaining in the anterior chamber, reducing its concentration. Even if the concentration remains unchanged, we still expect strong Doppler signal within the outflow vessels because the vessels are smaller than the glass capillary and thus experience less signal loss with depth. The experiment was carried out on cadaveric eyes, whose distal outflow pathway may have been blocked at certain sections due to coagulation or other factors. This may explain why Doppler signal was seen only in some outflow vessels but not others.

**Figure 5:**
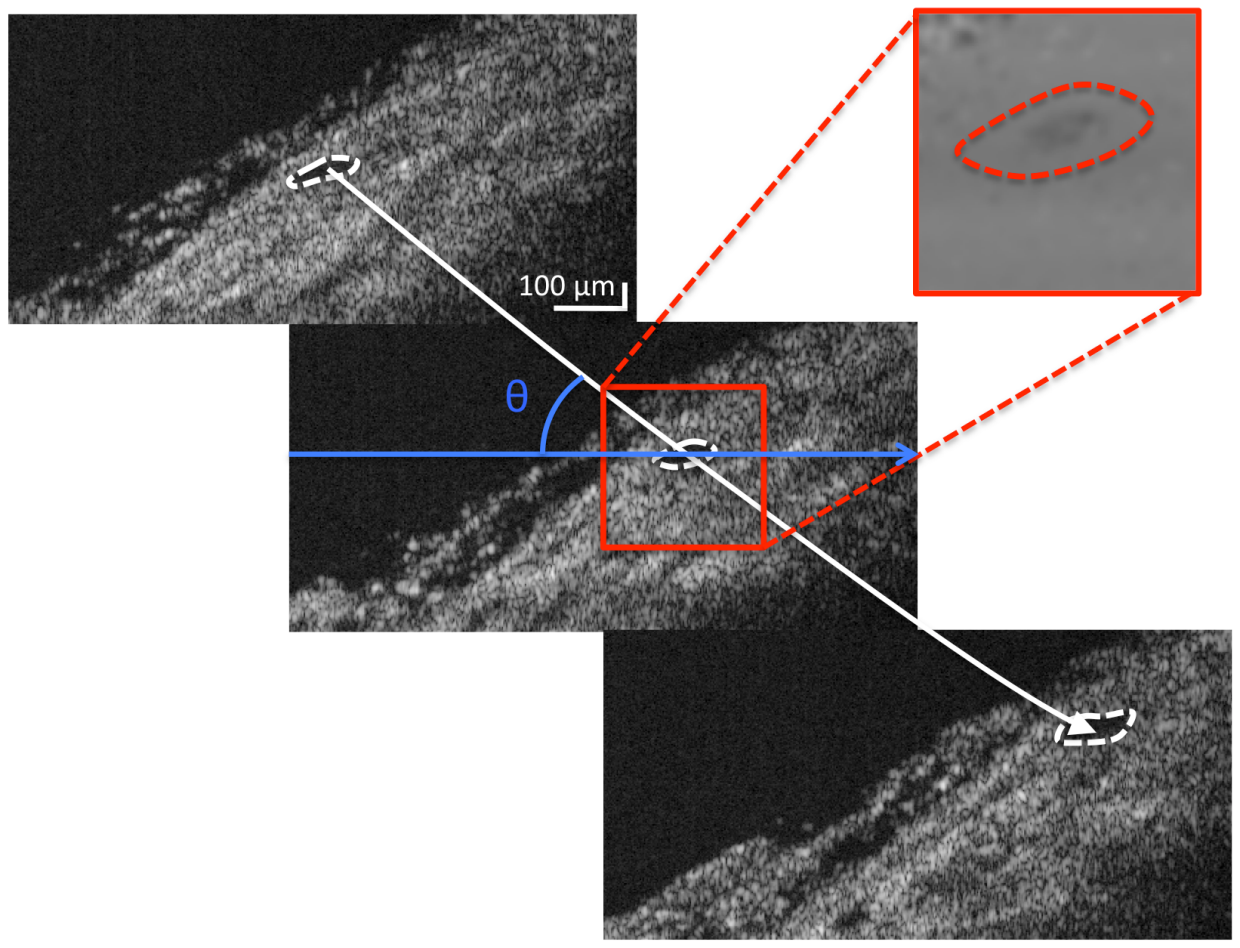
Determining volumetric flow. Adjacent B-scan images containing the vessels of interest are identified and marked (dashed white line) to determine the centroid. A line (white line) was fitted through the centroid to determine the angle of the vessel, θ, with respect to the OCT imaging beam (blue arrow). This allows us to integrate the flow within the Doppler information (red) and correct for the angle of flow with respect to the OCT scanning beam.

By integrating flow over the Doppler scans around the limbus, we were able to calculate volumetric aqueous flow in the eyes. The integration of the Doppler scans showed a slight overestimation of the total flow in both eyes. As seen in Figure 5, the directions of the vessels were found by fitting a line through the centroid of the vessels from adjacent scans. The angle between the OCT scanning beam and the line through the vessel centroid was used to determine the angle-adjusted flow rate. The overestimation might be due to the nearly perpendicular angle (60–80°) between the scanning beam and the aqueous outflow vessels. Therefore, even small differences in incident angle could have large effects on the flow calculations. Moreover, the vessels were traced in the 3D structural scans to determine their pathway. Highly tortuous vessels that showed as disconnected sections in the 3D scan could lead to an overestimation of the total flow if the same tortuous vessels is sampled multiple times. Furthermore, we cannot rule out that the Doppler scans did not account for the uveoscleral flow, which might further expand the reported difference total flow and the Doppler computed flow. However, this work provided proof of concept that GNRs could eventually be used to examine aqueous outflow.

While the experiments were conducted ex-vivo, a number of hurdles remain in the in-vivo use of GNRs. Current methods of inserting GNRs into the eye are invasive. A non-invasive delivery vehicle would help make GNRs more useful for scanning the outflow system in-vivo. Theoretically, the GNRs were small enough to pass through the cornea [16]. However, GNR eye drops were not used in this experiment because they would limit our ability to control the concentration within the eye. Another potential limitation of the GNR was the inflammatory response they induce in-vivo [15]. Further in-vivo studies are necessary to determine optimal coating for the GNRs to prevent such an inflammatory response. In addition, it is unknown how the excess GNRs are cleared, such as through macrophage induced phagocytosis, and in-vivo studies are necessary to insure that they do not permanently impede vision.

Improvements in Doppler imaging will also permit new possibilities for anterior segment imaging. Specifically, the use of swept-source lasers will permit superior imaging to the deeper regions of the angle, including schlemn's canal. Furthermore, advances in Doppler imaging, such as dual beam Doppler OCT [17], [18], permit Doppler measurements without the dependence on incidence angle of single beam systems.

To our knowledge, this represented the first use of OCT to visualize aqueous outflow. Previous studies on aqueous outflow primarily involved the use of fluoroscein [19]. However, fluorescein could only measure aqueous loss indirectly through decreasing fluorescein concentration. GNR enhanced OCT Doppler scanning might lead to increased understanding of how outflow changes in glaucomatous eyes. Moreover, the ability to relate structural findings with functional outflow measurements recorded simultaneously might be a useful tool in ocular research.

## Conclusion

This study demonstrated the use of gold nanorods (GNRs) for enhancing Doppler OCT. Doppler OCT measurements were validated in experimental control flow and demonstrated ex-vivo in intrascleral aqueous pathway in porcine eyes.

## Supporting Information

Table S1 **Laminar Flow.**
(DOCX)

Figure S1 **Spectrophotometry of GNR in various media.** Spectrophotometry of (1) mock aqueous humor (mock AH) alone, (2) 2×10^12^ gold nanorods/ml (GNR), (3) GNR mixed with distilled water and (4) GNR mixed with mock aqueous humor.
(TIFF)

Figure S2 **GNR flow during imaging.** Volume remaining in the reservoir over time for eye 1. A linear slope indicates a constant flow rate.
(TIFF)
