# Supplementary material for: Gold Nanorods as a Contrast Agent for Doppler Optical Coherence Tomography
Source: PLoS One. 2014 Mar 3;9(3):e90690. doi: 10.1371/journal.pone.0090690 (PMC3940929; doi:10.1371/journal.pone.0090690)
Supplement: Table S1 — Laminar Flow. (DOCX) [file pone.0090690.s001.docx]

**Table S1**

Using equation 1, we derived the theoretical v_max_ for a given recorded flow rate for laminar flow. The v_max_ can then be used in equation 2 to give the theoretical flow profile to be seen in a test tube. Based on a flow rate of 0.56 μL/s, the theoretical laminar flow profile is provided in equation 3.

Equation 1:

Equation 2:

Equation 3:

$$v(r)=-3.41(1-\frac{r^{2}}{0.32^{2}})$$
