# Supplementary figures and images for: Gold Nanorods as a Contrast Agent for Doppler Optical Coherence Tomography
Source: PLoS One. 2014 Mar 3;9(3):e90690. doi: 10.1371/journal.pone.0090690 (PMC3940929; doi:10.1371/journal.pone.0090690)

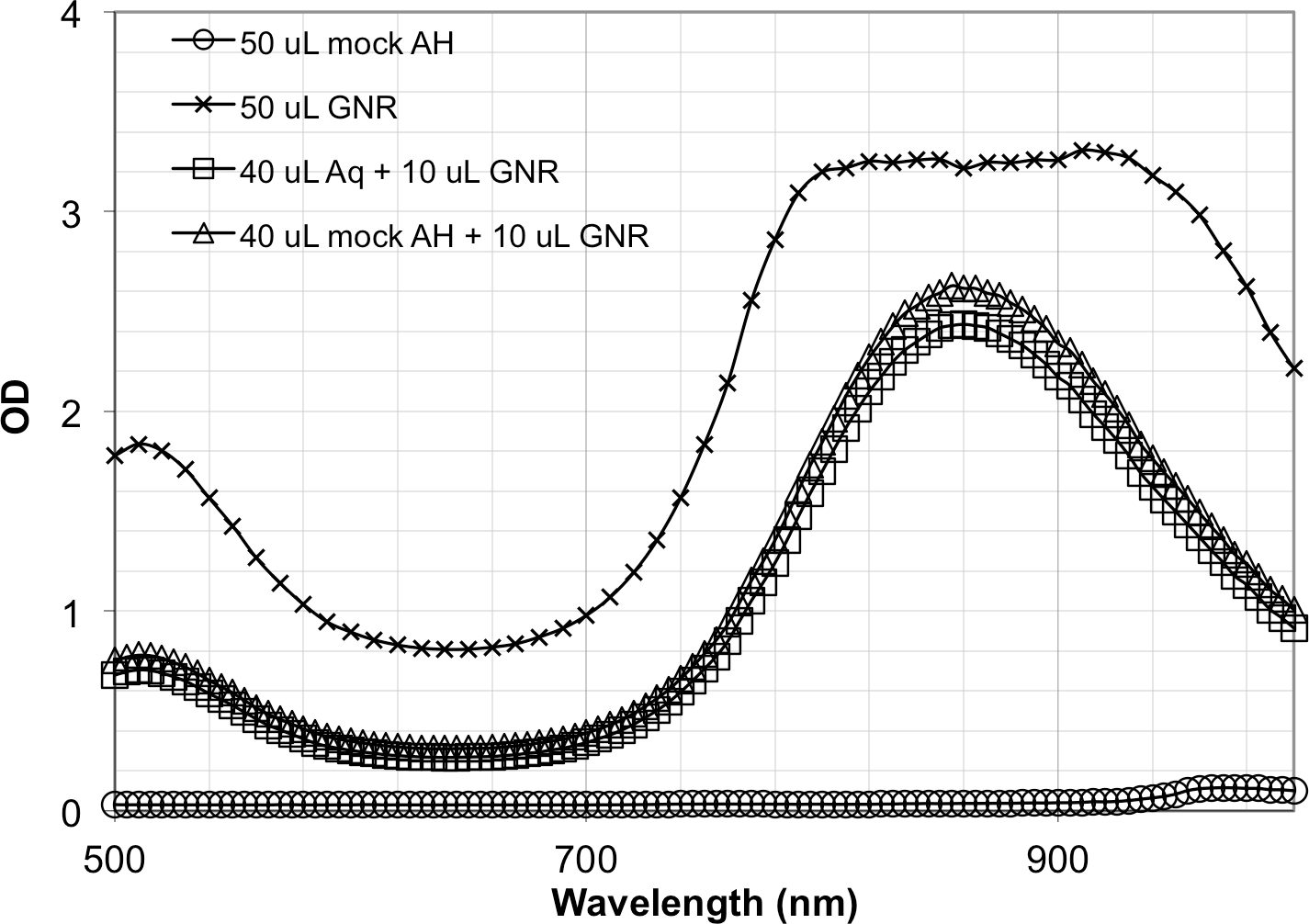

Supplement: Figure S1 — Spectrophotometry of GNR in various media. Spectrophotometry of (1) mock aqueous humor (mock AH) alone, (2) 2×10^12^ gold nanorods/ml (GNR), (3) GNR mixed with distilled water and (4) GNR mixed with mock aqueous humor. (TIFF) [file pone.0090690.s002.tiff]

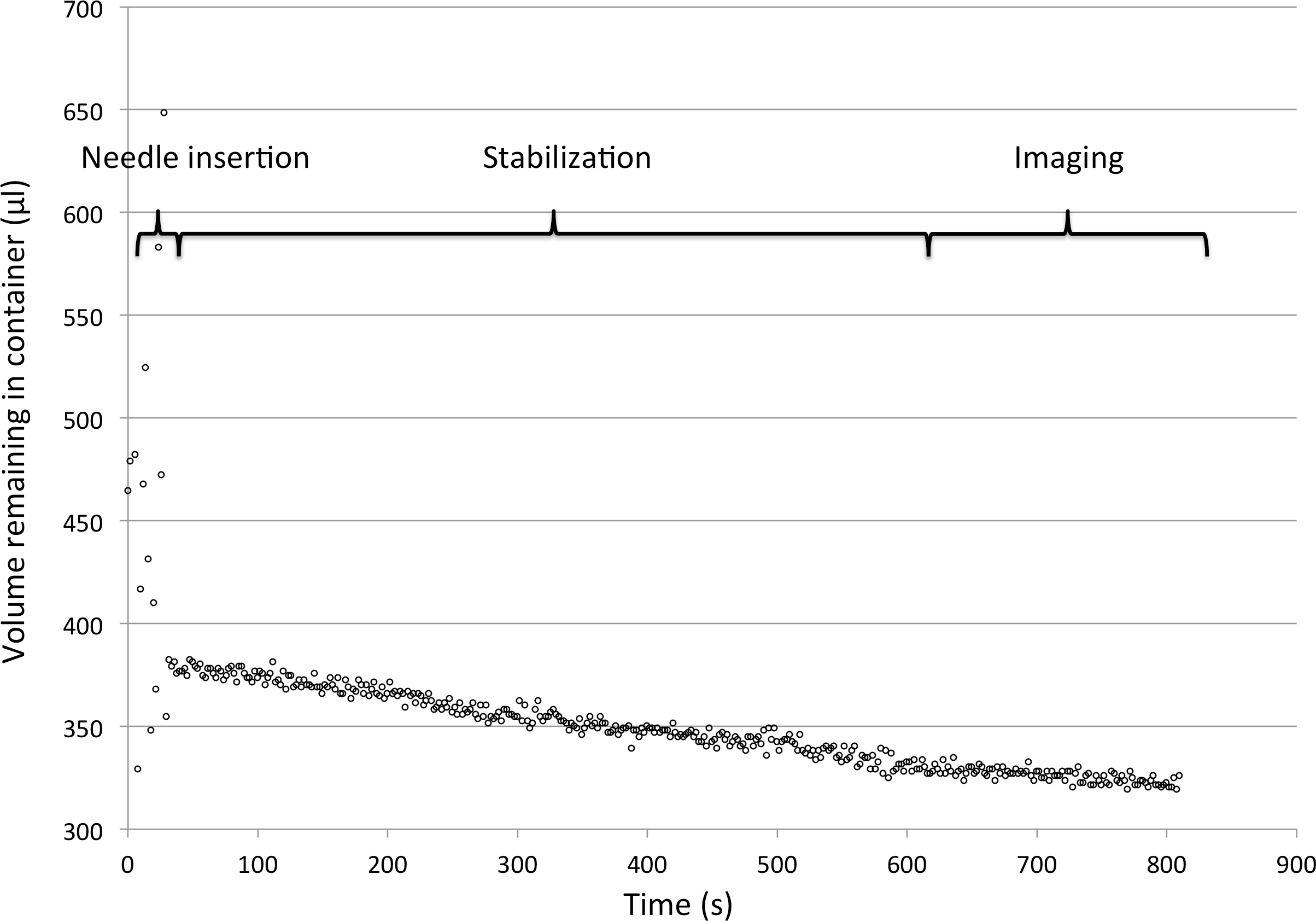

Supplement: Figure S2 — GNR flow during imaging. Volume remaining in the reservoir over time for eye 1. A linear slope indicates a constant flow rate. (TIFF) [file pone.0090690.s003.tiff]
